# Accidental Arthrotomy Causing Aseptic Monoarthritis Due to Agave Sap: A Case Report

**DOI:** 10.5811/cpcem.2021.4.51835

**Published:** 2021-05-06

**Authors:** Sam T. Ontiveros, Alicia B. Minns

**Affiliations:** University of California, San Diego, Department of Emergency Medicine, San Diego, California

**Keywords:** Agave, septic arthritis, inflammatory arthritis, plant

## Abstract

**Introduction:**

Aseptic inflammatory arthritis has been reported from thorns or cactus needles after inadvertent arthrotomy. Agave sap irritants may cause an aseptic inflammatory arthritis mimicking a septic joint.

**Case Report:**

A 27-year-old male presented with left knee pain and swelling two hours after suffering an accidental stab wound to his left lateral knee by an agave plant spine. Synovial fluid white blood cell count was 92,730 mm^3^ with 75% neutrophils and no crystals. Surgical washout was remarkable for turbid fluid and no foreign body. Synovial fluid and blood cultures remained without growth. At two-week follow-up, the patient had recovered.

**Conclusion:**

Penetrating injuries from agave thorns can cause an inflammatory arthritis that mimics septic arthritis.

## INTRODUCTION

Agave is a genus of flowering plants native to hot and arid regions. Most agave species have a sharp terminal spine and are very fibrous. *Agave Americana* is one of 300 species of agave and is native to the southwestern United States and Mexico.^1^ Contact with agave has previously been reported to cause both vesiculopapular and purpuric dermatitis. Causative agents are believed to be steroidal saponins and calcium oxalate crystals in agave sap.^1^ Aseptic inflammatory arthritis has been reported from foreign bodies such as thorns or cactus needles after inadvertent arthrotomy. Here we describe a case of aseptic inflammatory arthritis caused by agave sap irritants without foreign body that mimicked septic arthritis.

## CASE REPORT

**Image 1:**
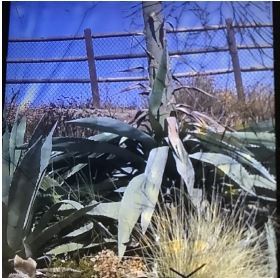
Photograph taken by patient of agave plant.

A 27-year male presented to the emergency department (ED) with malaise, left knee pain, and swelling two hours after suffering an accidental stab wound to his left lateral knee by an agave plant spine. The patient rapidly developed knee pain, swelling, erythema, and inability to range the joint, prompting his ED visit. He had no history of intravenous drug use, sexually transmitted infection, or current genitourinary complaints. On examination, he had a temperature of 97.3ºF, blood pressure 143/73 millimeters mercury, heart rate of 89 beats per minute, and oxygen saturation of 99% on room air. Exam revealed a warm, erythematous, and swollen joint with a range of motion limited by significant pain. He had a small puncture wound noted to the lateral aspect of his left knee. The patient provided a photograph of the actual plant for identification (Image 1).

**Image 2:**
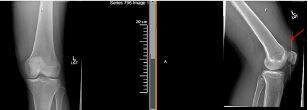
Radiograph of the left knee demonstrating a moderately sized effusion (arrow) without foreign body in a patient whose knee was punctured by an Agave plant spine.

Diagnostic testing included a white blood cell count (WBC) of 14 (1,000/cubic millimeters [mm^3^]),) erythrocyte sedimentation rate of 2 millimeters per hour (mm/hr) (normal 0–15 mm/hr), C-reactive protein of 2.2 milligrams per deciliter (mg/dL) (normal < 0.5mg/dL), and serum lactate was 2.6 millimoles per liter (mmol/L) (normal 0.5–2.0 mmol/L). The remainder of his complete blood count and basic metabolic panel was within normal limits. Radiograph of the knee demonstrated a moderately sized joint effusion without foreign bodies or bony abnormalities (Image 2). Arthrocentesis performed in the ED showed synovial fluid WBC was 92,730 mm^3^ with 75% neutrophils and no crystals. Gram stain was negative. The patient was taken for a left knee arthrotomy where approximately 100 cubic centimeters of purulent fluid was drained, and his knee was irrigated. No foreign body was found. Synovial fluid cultures (bacterial and fungal) and blood cultures remained without growth. Urine gonorrhea/chlamydia polymerase chain reaction testing was negative. Biopsy of the left knee synovium and fat demonstrated mild neutrophilic infiltrates and no fungal elements. Intravenous antibiotics (vancomycin and piperacillin/tazobactam) were started prior to incision and drainage; however, they were stopped on postoperative day 1. At a two-week follow-up appointment, he reported minimal residual discomfort that was treated with naproxen.

## DISCUSSION

We present a case of penetrating injury with an agave spine mimicking septic arthritis. Interestingly, no foreign body was found during operative washout, making this case unlike prior reported cases of monoarticular arthritis from plant injuries. There are published case reports describing plant injuries causing a monoarticular arthritis, such as plant thorn synovitis; however, in almost all cases, a foreign body was found during arthroscopy or arthrotomy.^2^,^3^ Salient features in these reported cases include the rapid onset of symptoms from time of injury and the presence of a penetrating wound, which were also present in our case. In many published cases, radiograph is of limited diagnostic benefit as a foreign body is not always seen. Imaging is helpful in excluding other etiologies of joint pain, especially if a history of plant injury is not provided.^2^,^4^ Formal or point-of-care ultrasonography was not performed in this case, but it has been demonstrated to be helpful in localizing foreign bodies not seen on plain radiography.^5^ Ultrasound guidance may also improve aspiration success during arthrocentesis.^6^

**CPC-EM Capsule**

What do we already know about this clinical entity?

*Exposure to Agave plants has been reported to cause skin irritation and rashes. Plant arthrotomies can lead to an inflammatory arthritis and a foreign body is often found during surgery.*

What makes this presentation of disease reportable?

*To our knowledge, accidental arthrotomy from an Agave plant leading to inflammatory arthritis has not been reported.*

What is the major learning point?

*The irritating sap of the Agave can cause an inflammatory arthritis that mimics septic arthritis if introduced into a joint.*

How might this improve emergency medicine practice?

*Although rare, physicians need to consider plant injury when evaluating patients with a rapidly developing monoarticular arthritis.*

As a general rule, the causative bacteria in most cases of septic arthritis are Gram-positive organisms such as *Staphylococcus aureus*, with increasing incidence of methicillin resistance. *Neisseria gonorrhoeae* usually occurs in younger adults, although these patients typically present with migratory polyarthritis, a pustular rash, and urethritis.^7^ While a rare cause of septic arthritis, Lyme arthritis is the most common manifestation of late-stage Lyme disease, and should be considered in patients who reside in endemic areas.^8^ Septic arthritis caused by plant injuries have been infrequently described, but should be considered when evaluating patients presenting with a monoarticular arthritis. *Pantoea agglomerans* is a Gram-negative bacterium belonging to the family Enterobacteriaceae, and can be found in human and animal feces and in plants.^9^ *P. agglomerans* is rare, but has been described in several cases of arthritis or synovitis caused by plant thorns, classically the palm tree thorn.^4^ Diagnosis is often delayed due to the low virulence of this organism and difficulty in culturing it.^9^

*Agave americana*, the causative plant in this case, has historically been used to create rope, vinegar, syrups, and fermented drinks, in addition to its contemporary use as a decorative plant.^1^ *A. americana* is more commonly known as the “century plant” because it was originally believed to only flower once every 100 years.^10^ *A. americana* contains calcium oxalate crystals and saponins, which have been reported to cause cutaneous reactions after contact, such as irritant dermatitis and vesiculopapular eruptions.^1^,^10^ Bundles (raphides) of sharp calcium oxalate crystals also occur in other plants such as *Dieffenbachia amoena* (a common house plant also known as dumb cane) and are notorious for causing irritation to skin and mucous membranes on contact.^11^ We postulate that in our case the severely irritating sap of *A. americana* caused an acute inflammatory arthritis when introduced into the joint.

## CONCLUSION

Although plant injuries to the joint are uncommon, it should be considered in cases of rapidly developing acute monoarticular arthritis. A history of penetrating injury from a plant is often overlooked. It is recommended that in the presence of a penetrating injury, arthroscopic washout or formal arthrotomy with inspection of the joint be pursued, because there are multiple published examples of recurrent episodes of joint sepsis/synovitis due to retained plant material.^3^ In our case a history of plant injury was provided, and the irritating sap of agave caused an inflammatory arthritis without foreign body.
